# The DRY Box and C-Terminal Domain of the Human Cytomegalovirus US27 Gene Product Play a Role in Promoting Cell Growth and Survival

**DOI:** 10.1371/journal.pone.0113427

**Published:** 2014-11-19

**Authors:** Carolyn C. Tu, Juliet V. Spencer

**Affiliations:** Department of Biology, University of San Francisco, San Francisco, California, United States of America; University of Regensburg, Germany

## Abstract

Human cytomegalovirus (HCMV) is a widespread pathogen that can lay dormant in healthy individuals and establish lifelong latent infection. This successful co-existence is facilitated by a number of viral gene products that manipulate host cellular functions and immune responses. Among these immunomodulatory genes are four G-protein coupled receptors (GPCRs) encoded by HCMV, designated US27, US28, UL33, and UL78. Studies have shown the US28 gene product to be a functional chemokine receptor that signals both constitutively and in a ligand-dependent manner, resulting in a wide range of cellular effects. In previous work, we have found that US27 expression results in at least two biological effects: enhanced CXCR4 signaling and increased in cellular proliferation in HEK293 cells. Here, we examined the involvement of two protein domains, the DRY box and the C-terminal intracellular domain (CTD) of US27, in mediating both cell proliferation and survival. While both domains were required for a proliferative effect, loss of either domain only moderately impacted cell survival, suggesting that US27 may interact with cell survival pathways through protein regions other than the DRY box and CTD. Quantitative RT-PCR arrays were used to profile changes in cellular gene expression in the HEK293-US27 cell line, and down-regulation of cell cycle regulators CDKN1A/p21/CIP1 (cyclin dependent kinase inhibitor 1A) and SESN (Sestrin2 or Hi95) was observed. These results indicate that increased cell proliferation due to US27 may be linked to suppression of negative growth regulators, and that these interactions require the DRY box and CTD.

## Introduction

Human cytomegalovirus (HCMV) is a member of the *Herpesviridae* family that manipulates the host immune system and establishes life-long latent infection [1]. HCMV is widespread in the general population, but infection is typically asymptomatic in immune competent individuals [2]. Transplant recipients and HIV patients, on the other hand, can suffer debilitating disease upon virus reactivation [3], [4]. In addition, during pregnancy, HCMV may be transmitted *in utero* to the developing fetus, resulting in severe congenital defects like deafness, blindness, mental retardation, and impaired motor function [5], [6].

Successful co-existence of HCMV with a healthy host is mediated to some extent by the production of viral proteins that mimic normal immune modulators like cytokines, chemokines, and chemokine receptors [7]. The US27 gene encodes a protein with seven transmembrane domains and similarity to the human chemokine receptor family of G-protein coupled receptors (GPCRs), including conserved cysteines in the extracellular loops and extensive glycosylation of the extracellular domain [8]. US27 also contains a DRY (aspartic acid, arginine, tyrosine) motif in the second intracellular loop that is critical in other GPCRs for activation of G proteins following ligand binding [9], as well as a di-leucine motif in the carboxy-terminal domain that mediates receptor endocytosis [10]. US27 is found in the envelope of the virus particle [8], but in virus-infected cells, the majority of the US27 protein is found in endosomes, the Golgi apparatus, and perinuclear compartments [11]. The US27 gene is non-essential for virus replication and mutants that lack US27 are replication competent [12]. However, US27 deletion mutant viruses are incapable of spreading via the extracellular route in endothelial cells [13], suggesting that US27 might play a role in virion assembly or egress.

Although no chemokine ligands for US27 have been identified, signaling activity of the human chemokine receptor CXCR4 is increased in the presence of US27 [14]. In addition, cells expressing US27 demonstrate increased rates of cell proliferation and DNA synthesis [15]. Here, we further investigated the mechanism for this proliferative effect by examining the requirement for the DRY box motif and the carboxy-terminal domain (CTD) of US27 in cell growth and resistance to apoptotic stimuli.

## Materials and Methods

### Cells

Human embryonic kidney (HEK) 293 cells were grown in Eagle's minimal essential media (MEM) with 10% fetal bovine serum (Cellgro, Herndon, VA) in a humidified incubator at 37°C and 5% CO_2_ atmosphere. Stable HEK293 cell lines expressing 3XFLAG-tagged HCMV US27, US28, or human CXCR3 were generated previously and cultivated under the same conditions [10]. In addition, stable cell lines expressing 3XFLAG-tagged US27-DAY (R128A) and US27/XR3CT were also cultured as above [14].

HEK293 or HeLa cells were maintained as above, then seeded in white 96-well plates at a density of 1×10^4^ cells per well and transiently transfected 24 hours later with the indicated pEGFP expression vectors using Fugene transfection reagent (Roche Biosciences, Basel, Switzerland) at a ratio of 3∶1 (µl Fugene: µg plasmid DNA per manufacturer's instructions). Transfection efficiency was 40–50% as determined via flow cytometry.

### Western Blotting

Cells were seeded into 100 mm dishes at a density of 8×10^5^ cells and lysed in the dish after 48 hours via the addition of lysis buffer (150 mM NaCl, 20 mM HEPES, 0.5% Cymal-5, 1 mM NaOV_4_, 1 mM EDTA, 0.1% NaN_3_ and 4 M urea). The lysate was freeze-thawed three times, clarified via centrifugation at 14,000 rpm for 15 minutes at 4°C, followed by addition of loading dye. Samples were separated using SDS-PAGE, and then proteins were transferred to a nitrocellulose filter then blocked in Tris-buffered saline containing 0.1% Tween-20 (TBS-T) +5% milk for one hour. The membranes were incubated with either anti-p21 monoclonal antibody (Santa Cruz Biotechnology, Santa Cruz, CA), anti-sestrin or anti-MAPK rabbit polyclonal antiserum (Cell Signaling Technology, Danvers, MA) as indicated according to the manufacturer's instructions. For anti-FLAG M2 western blotting, cells were seeded as indicated above and scraped 48 hours later in cold RIPA buffer (25 mM Tris-HCl pH 7.6, 150 mM NaCl, 1% NP-40, 1% sodium deoxycholate, 0.1% SDS), followed by PBS wash, and resuspension of pellet with 100 µl of cold RIPA buffer. Cell lysates were agitated every 15 minutes for one hour, sonicated, and centrifuged at 14,000 rpm at 4°C for 15 minutes. Clarified cell lysates were then transferred into eppendorf tubes. After the addition of 4× Laemmli buffer (Bio-Rad) and 10× sample reducing agent (Life Technologies), cell lysates were heated at 42°C for 10 minutes. Samples were then separated using SDS-PAGE and transferred onto a nitrocellulose membrane as described above, followed by overnight incubation at 4°C with anti-FLAG M2 monoclonal antibody (Sigma Aldrich, St. Louis, MO) at a 1∶7500 dilution. For detection, membrane was incubated with appropriate alkaline phosphatase-conjugated secondary antibody. Bands were visualized via the addition of Western Blue Substrate reagent (Promega, Madison, WI).

### Immunofluorescence Microscopy

HEK293 and stable cells were seeded in six-well dishes containing FBS coated glass coverslips at a density of 1.5×10^5^ cells per well and then incubated for 48 hours at 37°C. Cell monolayers were washed with PBS, fixed with 4% paraformaldehyde, then permeabilized with 0.2% (w/v) Triton X-100. Cells were then blocked with PBS +10% FBS for one hour at 37°C and stained with anti-FLAG M2 antibody (Sigma-Aldrich) at a 1∶1000 dilution for one hour at 37°C. Following three PBS washes, the coverslips were incubated with Alexa Fluor 514 Goat Anti-Mouse (Life Technologies) at a 1∶500 dilution for one hour at 37°C, washed again, and then mounted on a glass slide using Prolong Gold anti-fade reagent with DAPI (Invitrogen, Carlsbad, CA). For cell surface receptor staining only, the protocol was followed as described above but cells were not permeabilized. All images were acquired using a Zeiss LSM700 laser scanning confocal fluorescence microscope and Zen Black software (Carl Zeiss, Inc., Oberkochen, Germany).

### Proliferation Assays

Cells were seeded in white 96-well dishes at a density of 5×10^3^ cells per well. Cell growth was measured at indicated time points via the addition of CellTiter-Glo reagent (Promega) followed by luminometry according to manufacturer's instructions. Bromodeoxyuridine (BrdU) incorporation was assayed on cells seeded as above using the BrdU ELISA kit (Roche Bioscience, South San Francisco, CA). For both assays, each condition was performed in triplicate within the assay, and three independent replicates of each experiment were performed. For cell counts, cells were seeded into 6-well dishes at 2×10^5^ cells per well, harvested via trypsinization at the indicated times, and then 3×10 µl aliquots were analyzed from each well at the indicated times using the BD Accuri C6 Flow Cytometer (Becton Dickinson, San Jose, CA). Three independent experiments assessing cell counts were performed.

### Apoptosis Assays

Cells were seeded into 6-well dishes and treated with 10 µM etoposide (Sigma-Aldrich) for 48 hours, then harvested and stained with Annexin V and propidium iodide using the TACS Annexin V-FITC Staining Kit (Trevigen, Gaithersburg, MD) before analysis by flow cytometry. In addition, cells were seeded into white 96 well plates at a density of 5×10^3^ cells per well in the presence of 10 µM etoposide, and cell viability determined at indicated time points via the addition of CellTiter-Glo reagent as above. For transiently transfected HEK293 and HeLa cells, 10 µM etoposide was added six hours post-transfection. In all cases, each condition was performed in triplicate within the assay, and three independent experiments were performed.

### PCR

RNA from HEK293 or stable cell lines that were 70–80% confluent in a T75 flask was extracted using RNEasy Midi Kit (Qiagen, Valencia, CA), cDNA was prepared using the RT2 First Strand Kit (Qiagen), and then diluted cDNA was mixed with the RT2 SYBR green master mix (Qiagen) according to the manufacturer's instructions and loaded into the Human p53 Signaling Pathway RT2-PCR Profiler Array (SABiosciences, Valencia, CA). Real-Time PCR was performed using the CFX96 (BioRad, Hercules, CA) by heating to 95°C for 10 minutes followed by 40 cycles of 95°C for 15 seconds and 60°C for 1 minute. Data were analyzed using the ΔΔCt method according to the SABiosciences web portal (www.SABiosciences.com/pcrarray.dataanalysis.php) as described previously [15] and are provided as Supplementary Data. The same threshold value was used across all plates in the same data analysis to ensure accurate reading of quality controls. The data were normalized across all plates to the following housekeeping genes: beta-2-microglobulin (B2M), hypoxanthine phosphoribosyltransferase 1 (HPRT1), ribosomal protein L13a (RPL13A), glyceraldehyde-3-phosphate dehydrogenase (GAPDH), and beta actin (ACTB). Controls for genomic DNA contamination, RNA quality, and PCR performance were all in the manufacturer's recommended ranges. The fold change values from three biological replicates of the PCR array were analyzed in comparison to either HEK293 cells or 293-CXCR3 cells as indicated. For RT-PCR, RNA from HEK293 or stable cell lines was extracted using RNeasy Midi Kit (Qiagen, Valencia, CA). cDNA was prepared using the iscript cDNA synthesis kit (Bio-Rad). For PCR reactions, each contained cDNA template, primers, dNTP mix, Ex-Taq buffer, and Ex-Taq polymerase (Clontech, Mountain View, California) in a final volume of 30 µl. The gene specific primers for Sestrin-2 were 5′ – CCTGCACCCTGACTACTTTAC – 3′ (forward) and 5′ – CCATGGTCTTCCCAGGTATAATC – 3′ (reverse) and for β-actin specific primers, 5′ – AAGAGAGGCATCCTCACC – 3′ (forward) and 5′ – TACATGGCTGGGTGTTG – 3′ (reverse). The PCR reaction underwent the following protocol on a MyCycler Thermal Cycler (Bio-Rad): 95°C for 1 min followed by 35 cycles of 94°C for 1 min, 55°C for 1 min, 72°C for 1 min, followed by one cycle of 72°C for 10 min, and a final hold at 4°C. The PCR products were visualized on a 1% agarose gel.

## Results and Discussion

**Figure 1:**
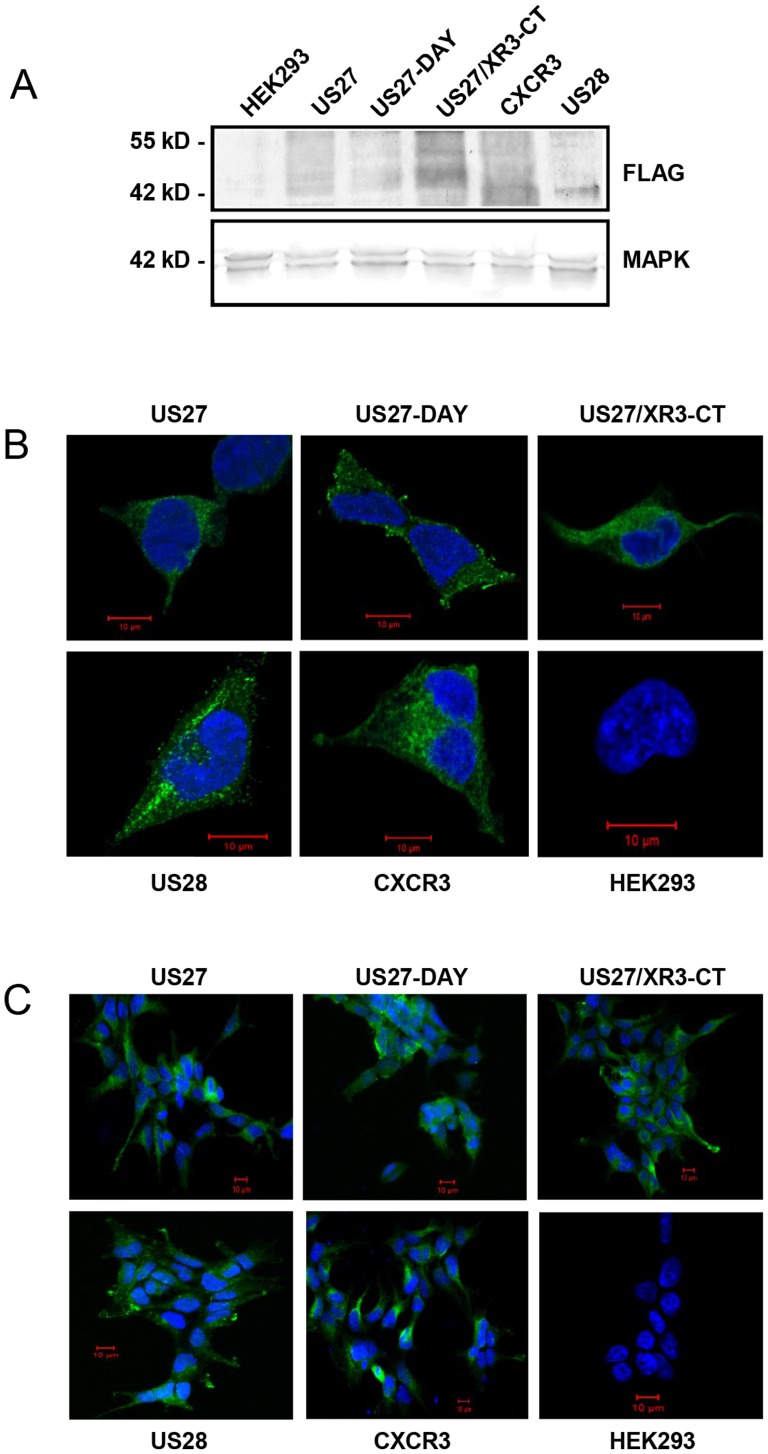
Stable cell lines express HCMV US27 or US27 mutants. A) Cell lysates were separated by SDS-PAGE and then immunoblotted using anti-FLAG M2 antibody or anti-MAPK as a control. (B) Immunofluorescence staining of fixed, permeabilized cells with anti-FLAG M2 antibody followed by Alexa Fluor 514-conjugated mouse secondary. Optical sectioning was performed and one slice of the Z-stack is shown. (C) Cell surface staining with anti-FLAG M2 antibody on non-permeabilized cells, followed by Alexa Fluor 514 secondary antibody.

The US27 gene product has been shown to promote an increase in cell proliferation when compared to cells expressing US28 or other cellular chemokine receptors like CXCR3 [15]. Here, we investigated whether specific domains of US27 are necessary to mediate this proliferative effect by making use of two mutants available in our laboratory. First, we examined the requirement for the DRY box motif that is known to play a role in signaling for many GPCRs [9] by using a stable HEK293 cell line expressing US27-DAY, which contains a single amino acid substitution of arginine 128 to alanine (R128A). We also explored the requirement for the intracellular carboxy-terminal domain (CTD) using both a stable HEK293 cell line expressing US27/XR3CT, a chimeric receptor that has the CTD of CXCR3 in place of the CTD from US27, as described previously [10], and a mutant lacking the CTD, US27ΔCT. As shown in Figure 1, wild-type US27 and the US27 mutants were expressed at comparable levels. Cell lysates were immunoblotted with antibody directed against the N-terminal 3XFLAG tag, and the expected broad band of 45–50 kD for the US27 protein, which is heavily glycosylated, was detected (Fig. 1A). Comparable bands were noted for both the US27-DAY and US27/XR3CT receptors, demonstrating that these changes do not adversely affect protein expression. US28 and CXCR3, an HCMV and a human chemokine receptor, respectively, serve as controls in several subsequent experiments and were also detected at the expected sizes of 44 kD and 42 kD. No 3XFLAG labeled proteins were detected in the parent HEK293 cell line. Each receptor was also viewed by immunofluorescence microscopy, and the expression level for the US27-DAY and US27/XR3CT proteins was similar to that of wild-type US27, US28, and CXCR3 (Fig. 1B). To confirm that the mutations in US27 did not alter receptor trafficking to the surface, cells were fixed but not permeabilized, then incubated with anti-FLAG antibody. As shown in Fig. 1C, the level of staining was comparable between US27 and the mutants, indicating that these receptors are present on the cell surface and that the mutations in US27 have no effect on receptor recycling.

**Figure 2:**
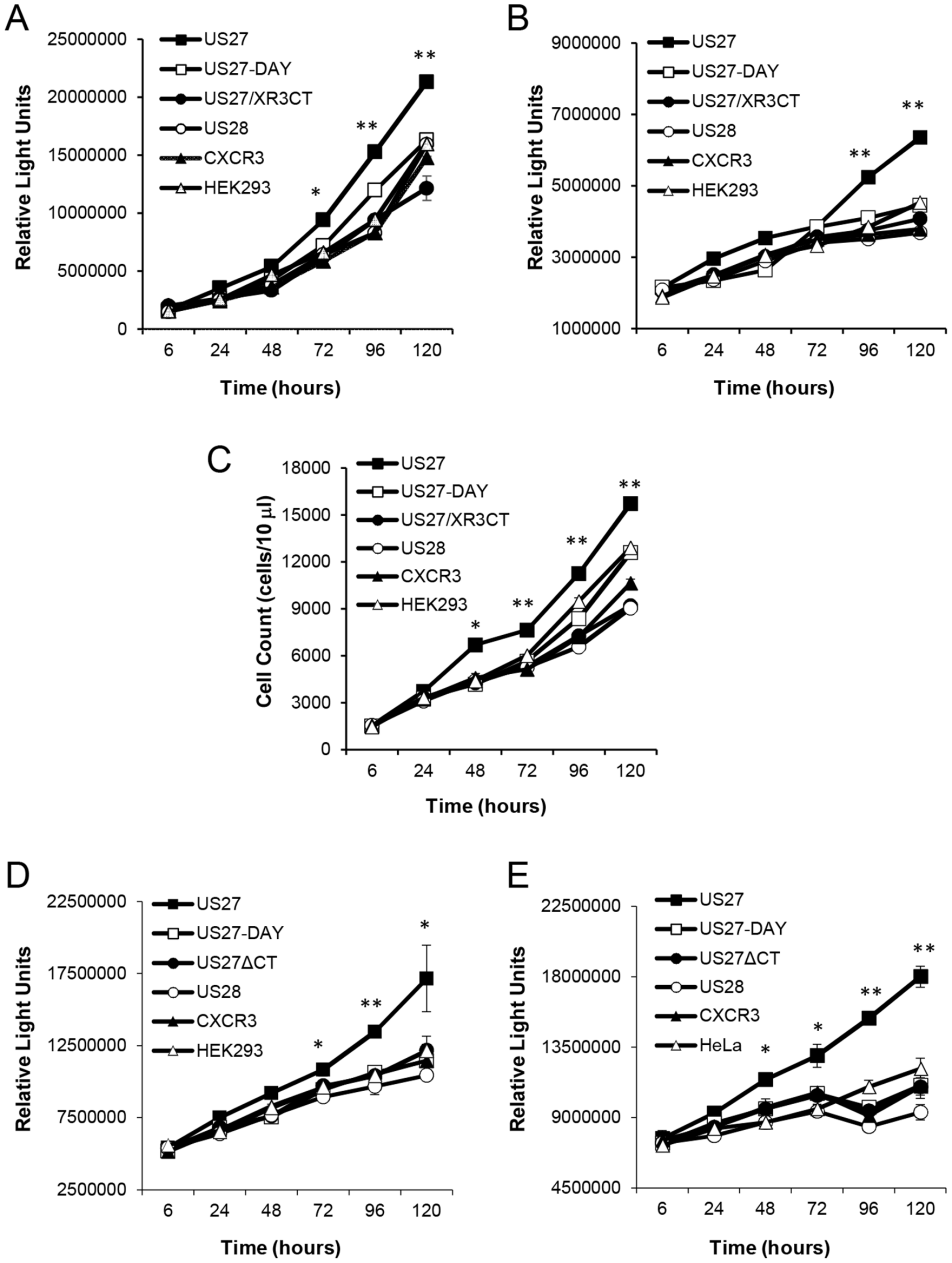
Expression of US27 conveys a proliferative advantage. A) Cells were seeded into 96-well plates at a density of 5×10^3^ cells per well and cell number was monitored via the addition of CellTiter-Glo reagent at the indicated time points. B) The rate of DNA synthesis for each cell line was measured using bromodeoxyuridine (BrdU) incorporation and luminometry. C) Cells were seeded in 6-well dishes at 2×10^5^ cells per well, then harvested with trypsin and counted via flow cytometry at the indicated time points. (D) HEK293 and (E) HeLa cells were seeded into 96-well plates at a density of 1×10^4^ cells per well and transfected 24 hours later with the indicated pEGFP expression vector at a ratio of 3∶1 (µl Fugene: µg plasmid DNA), cell number was monitored via the addition of CellTiter-Glo reagent at the indicated time points. Error bars represent standard error of three triplicate data points within one experiment. These results are representative of three independent experiments. ** indicates p<0.001, * indicates p<0.05 by Student t-test.

In order to examine the impact of these mutations on the ability of US27 to enhance cell proliferation, growth of the stable cell lines was monitored over time. Cell viability was evaluated using a substrate for total cellular ATP, reflecting the number of metabolically active cells in the well (Fig. 2A). Cells expressing wild-type US27 exhibited ATP levels that were higher than any of the other cell lines, which included the parent HEK293 cells, cells expressing US28, CXCR3, or the US27 mutants. Cells expressing US27-DAY and US27/XR3CT were healthy and exhibited growth, but the ATP levels were more similar to control cells than US27-expressing cells. There was no evidence that either mutation resulted in a protein that facilitated the same growth advantage as wild-type US27.

Cells expressing US27 mutants were further investigated using BrdU incorporation to determine whether there was any effect on the rate of DNA synthesis (Fig. 2B). While cells expressing wild-type US27 exhibited a marked increase in the rate of DNA synthesis relative to control cells, the US27-DAY and US27/XR3CT cell lines had a rate of DNA synthesis that was on par with the control cells. In addition, standard cell counts confirmed that only US27-expressing cells, and not the US27 mutant-expressing cell lines, contained higher cell numbers than controls when counted at the indicated times (Fig. 2C). While we have previously shown that US27 conveys increased proliferative properties in a variety of cell types [15], we wanted to examine the US27 mutants in a different cell type. Both HEK293 and HeLa cells were transiently transfected using EFGP-tagged US27, US27-DAY, or US27ΔCT constructs, and cell growth monitored (Fig. 2D, E). In both cell types, US27 cells grew at a significantly higher rate than cells expressing either mutant receptor or the controls. These results suggest that both the DRY box and the CTD of US27 may be required for stimulating cell proliferation.

**Figure 3:**
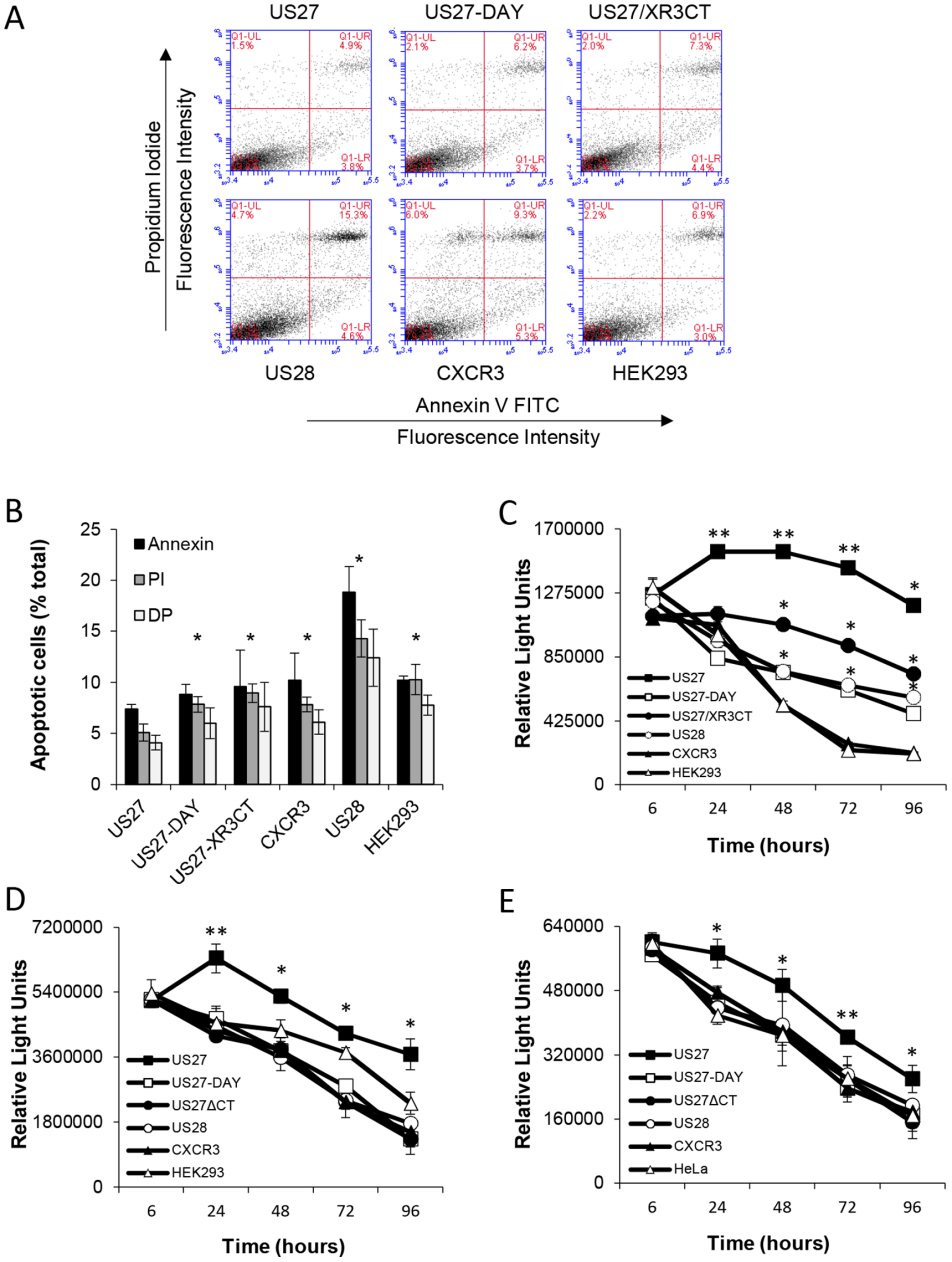
HEK293 cells expressing wild type US27 are more resistant to apoptosis. Cells were treated with 10 µM etoposide to induce apoptosis. A) After 48 hours, cells were stained with propidium iodide and Annexin V then analyzed by flow cytometry. B) The average percent positive cells for Annexin V, propidium iodide (PI) or both (double positive, DP) from three independent experiments, as indicated in A above. * indicates p<0.01 by Student t-test compared to US27-cells. Overall cell viability was measured at the indicated time points via the addition of CellTiter-Glo reagent and total luminescence quantified in C) stable HEK293 cell lines, and in transiently transfected D) HEK293 cells and E) HeLa cells following etoposide treatment. Error bars represent standard error of three triplicate data points within one experiment. These results are representative of three independent experiments. ** indicates p<0.001, * indicates p<0.05 by Student t-test. In C, pairwise comparisons were performed between US27/XR3CT and US27, US28, and HEK293 and between US27-DAY and US27 and HEK293.

Next we investigated whether US27 could protect cells from apoptosis. Cells were treated with etoposide to block topoisomerase function, thereby impeding DNA replication and leading to induction of the apoptotic cascade, which was detected via flow cytometry with Annexin V and propidium iodide staining. As shown in Figure 3A, cells expressing US27 had the lowest levels of apoptosis, with only 4.9% of the population staining double positive (DP) for Annexin V and propidium iodide after 48 hours. In contrast there were 15.3% DP cells in the US28 cell cultures, which is not surprising since this receptor has been reported to induce apoptosis when expressed in a variety of cell types [16]. Cultures expressing US27-DAY had 6.2% DP, which was comparable to the control HEK293 cell line (6.9%), and the US27/XR3CT cultures had 7.3% DP cells. Overall, US27-expressing cells had the lowest rate of etoposide-induced apoptosis (Fig. 3B), while cells expressing the US27 mutants responded more like the control cells, indicating that the DRY box and CTD may help mediate cell survival.

When cell viability was monitored over time, US27-expressing cells exhibited significantly greater viability than the other cells types (Fig 3C). Surprisingly, US27/XR3CT cells also had greater viability than most other cultures from 48 to 96 hours. The viability of cells expressing US27/XR3CT was significantly higher than US28 or control HEK293 cells, but significantly lower than US27-expressing cells, suggesting that this mutant may still be able to either partially block apoptosis or stimulate survival pathways, conferring a moderately protective effect. While US28 cells exhibited the highest levels of apoptosis at 48 hrs (Fig. 3A), the death rate in these cultures at later time points was significantly lower than in control HEK293 or CXCR3-expressing cells (Fig. 3B). The overall viability in the US27-DAY cultures was almost identical to that of US28 and also significantly lower than in control cells, indicating that this mutant might also retain some ability to protect cells from programmed cell death. Thus, unlike the proliferative ability of US27, which was completely abrogated by mutation of the DRY box and CTD, the ability to protect from etoposide-induced death was only partially diminished by these mutations. Given that the protection was less evident at the earliest time points, it may be that mutations delayed the activation of survival pathways by US27, but ultimately the US27-DAY and US27/XR3CT proteins were still able to convey anti-apoptotic effects to the cell.

In order to further explore the effect of the US27, US27-DAY, and US27/XR3CT on cell survival, quantitative PCR array analysis was performed on the stable cell lines. RNA was extracted from each cell type and expression levels of 84 genes involved in the p53 signaling pathway were compared to the parent HEK293 cells. As shown in Table 1, only a small subset of genes exhibited a fold change of 1.5 or higher that was statistically significant. Some genes were found to be down-regulated in all five cell lines stably expressing viral or human GPCRs, like CDKN1A, or p21, the cyclin-dependent kinase inhibitor. Although cells expressing US27 had the greatest fold change decrease (−2.41), the overall trend was that CXCR3, US28, and the US27 mutants all caused down-regulation of CDKN1A, suggesting that overexpression of any GPCRs might affect the p21 gene. Other genes, like BTG2 and BAI1, were specifically down-regulated in cells expressing the viral GPCRs but not in cells expressing CXCR3. For BTG2, (also known as NGF-inducible anti-proliferative protein PC3 or NGF-inducible protein TIS21), which is a p53-induced transcriptional co-regulator of cell cycle progression [17], cells expressing US27 exhibited a −2.05-fold change compared the control HEK293 cells, while cells with the US27-DAY mutant had only a −1.47-fold change and cells expressing US27/XR3CT had a modest 1.17-fold decrease in expression. This suggests that US27 impacts the expression of BTG2 and that this effect is mediated to some extent by the DRY box and to an even greater extent by the CTD of US27, which may interact with intracellular signaling proteins. For BAI1, brain-specific angiogenesis inhibitor 1, in the adhesion GPCR family that is regulated by p53 [18], the opposite effect was observed. Cells expressing US27 were found to have a −3.47 fold decrease in expression, compared to −1.02 for US27-DAY and −2.03 for US27/XR3CT, suggesting that the DRY box is critical for down-regulation of BAI1 by US27, while the CTD may play a less significant role in mediating that effect.

**Table 1:** p53 Pathway^1^ Gene Expression Analysis.

				Fold Change vs. HEK293				
Unigene	Refseq	Symbol	Description	US27	US27-DAY	US27/XR3CT	US28	CXCR3
Hs.519162	NM_006763	BTG2	BTG family, member 2	−2.05	−1.47	−1.17	−1.63	−1.12
Hs.80409	NM_001924	GADD45A	Growth arrest and DNA-damage-inducible, alpha	−1.34	1.35	−1.03	1.22	1.35
Hs.20930	NM_020418	PCBP4	Poly(rC) binding protein 4	−1.59	1.56	1.29	−1.16	1.25
Hs.241570	NM_000594	TNF	Tumor necrosis factor	−1.33	1.07	1.62	4.41	1.41
Hs.521456	NM_003842	TNFRSF10B	Tumor necrosis factor receptor superfamily, member 10b	−1.88	1.09	1.02	−1.05	1.14
Hs.194654	NM_001702	BAI1	Brain-specific angiogenesis inhibitor 1	−3.47	−1.02	−2.03	−2.86	−1.17
Hs.208124	NM_000125	ESR1	Estrogen receptor 1	−2.13	3.02	5.18	−1.76	−1.60
Hs.469543	NM_031459	SESN2	Sestrin 2	−1.87	1.27	1.49	−1.15	2.14
Hs.591980	NM_000378	WT1	Wilms tumor 1	−1.17	−4.49	−1.09	−1.84	−1.06
Hs.370771	NM_000389	CDKN1A	Cyclin-dependent kinase inhibitor 1A (p21, Cip1)	−2.41	−1.32	−1.51	−1.20	−1.64
Hs.326035	NM_001964	EGR1	Early growth response 1	−1.78	−1.33	−1.73	−1.05	1.24
Hs.93177	NM_002176	IFNB1	Interferon, beta 1, fibroblast	−1.18	1.05	2.00	−1.12	1.59
Hs.406266	NM_000189	HK2	Hexokinase 2	−1.46	1.07	1.04	−1.49	1.35

1 The complete list of genes analyzed using the Human p53 Signaling Pathway RT2 Profiler PCR Array (SABiosciences, Valencia, CA) includes: APAF1, ATM, ATR BAI1, BAX, BCL2, BCL2A1, BID, BIRC5, BRCA1, BRCA2, BTG2, CASP2, CASP9, CCNB2, CCNE2, CCNG2, CCNH, CDK1, CDC25A, CDC25C, CDK4, CDKN1A, CDKN2A, CHEK1, CHEK2, CRADD, DNMT1, E2F1, E2F3, EGR1, EI24, ESR1, FADD, FASLG, FOXO3, GADD45A, GML, HDAC1, HK2, IFNB1, IGF1R, IL6, JUN, KRAS, PIDD, MCL1, MDM2, MDM4, MLH1

MSH2, MYC, MYOD1, NF1, NFKB1, TP53AIP1, KAT2B, PCBP4, PCNA, PPM1D, PRC1, PRKCA, PTEN, PTTG1, RB1, RELA, RPRM, SESN1, SESN2, SIAH1, SIRT1, STAT1, TADA3, TNF, TNFRSF10B, TNFRSF10, TP53, TP53BP2, TP73, TP63, TRAF2, TSC1, WT1, XRCC5.

**Figure 4:**
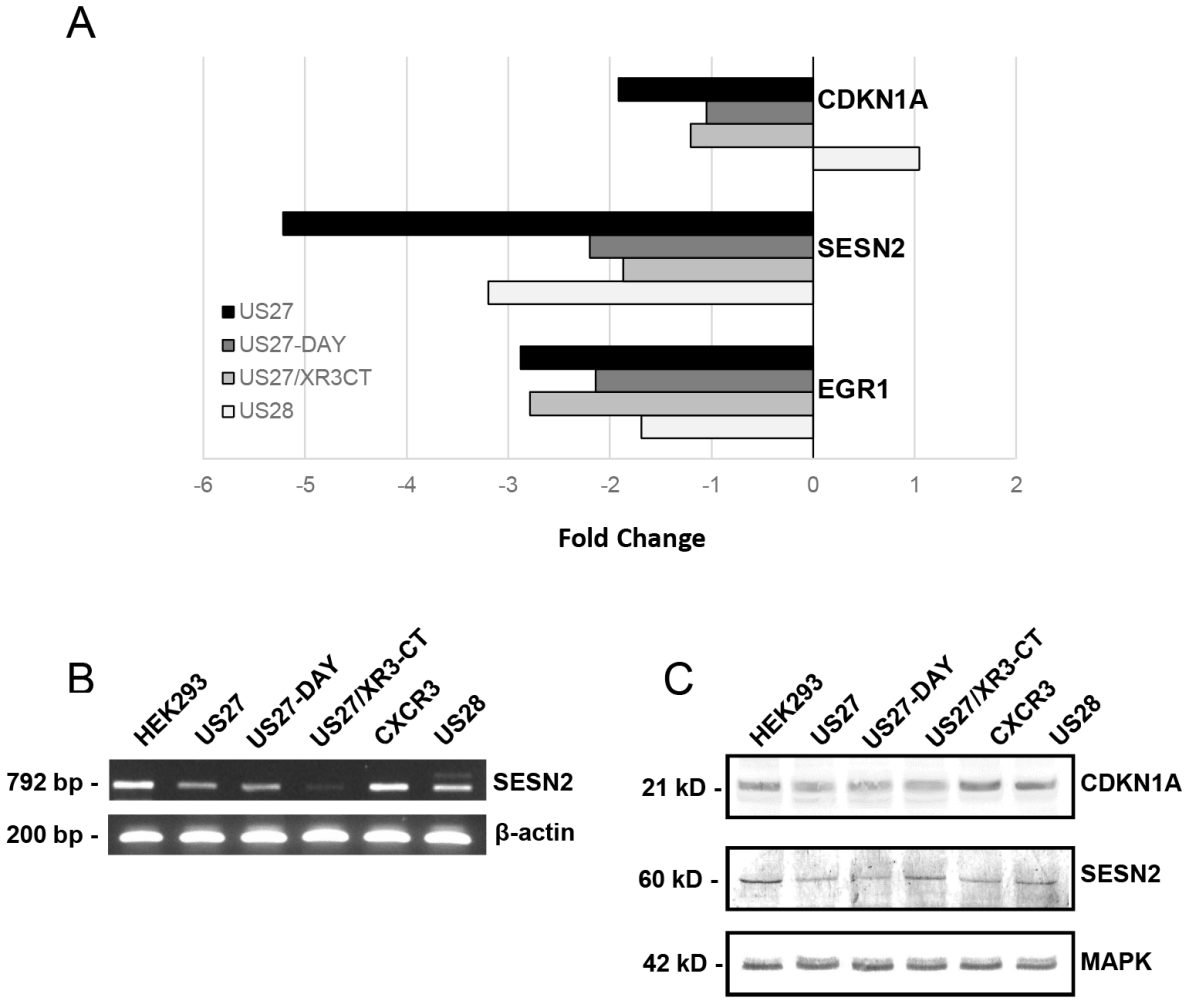
HCMV US27 correlates with decreased expression of p21 and Sestrin-2. A) Fold changes in gene expression compared to levels in cells expressing CXCR3. B) RNA was harvested from each stable cell line, reverse transcribed, then gene specific primers were used to amplify either Sestrin-2 or β-actin. C) Cell lysates were separated by SDS-PAGE and immunoblotted with antibodies directed against p21, sestrin-2, or MAPK as indicated.

For some genes, the changes in gene expression conveyed by any of the viral GPCRs were quite distinct from that of the human GPCR CXCR3. Thus, the fold changes were also calculated using the values from 293-CXCR3 cells as a baseline (Table 2). As shown in Figure 4A, both EGR1 and SESN2 were significantly down-regulated for all four viral GPCR. For SESN2, this pattern was also evident at a range of time points of cell cultivation (24 hours, Fig 4B), and at the protein level. Western blotting revealed decreased levels of Sestrin-2 (also known as Hi95) in cell lysates expressing US27 and US27-DAY compared to parent HEK293 or CXCR3-expressing cells (Fig. 4C), although for US27/XR3CT, the changes in RNA level did not correlate as clearly with protein level. Sestrin-2 has been identified as a stress sensor that functions in a p53-independent manner, possibly through the PI3K/Akt signaling pathway [19], [20]. Although Sestrin-2 is generally considered a pro-survival factor, our results suggest that the protein is down-regulated by US27, indicating that the signaling pathways involved may be complex and multi-functional.

**Table 2:** p53 Pathway^1^ Gene Expression Analysis II.

				Fold Change vs. 293-CXCR3			
Unigene	Refseq	Symbol	Description	US27	US27-DAY	US27/XR3CT	US28
Hs.519162	NM_006763	BTG2	BTG family, member 2	−2.38	1.26	−1.36	−1.89
Hs.80409	NM_001924	GADD45A	Growth arrest and DNA-damage-inducible, alpha	−2.36	−1.3	−1.81	−1.44
Hs.20930	NM_020418	PCBP4	Poly(rC) binding protein 4	−2.56	−1.04	−1.25	−1.87
Hs.241570	NM_000594	TNF	Tumor necrosis factor	−2.45	−1.72	−1.14	2.39
Hs.521456	NM_003842	TNFRSF10B	Tumor necrosis factor receptor superfamily, member 10b	−2.8	−1.36	−1.46	−1.56
Hs.194654	NM_001702	BAI1	Brain-specific angiogenesis inhibitor 1	−3.89	−1.15	−2.28	−3.2
Hs.208124	NM_000125	ESR1	Estrogen receptor 1	−1.74	3.7	6.35	−1.75
Hs.469543	NM_031459	SESN2	Sestrin 2	−5.22	−2.2	−1.87	−3.2
Hs.591980	NM_000378	WT1	Wilms tumor 1	−1.44	−5.54	−1.35	−2.76
Hs.370771	NM_000389	CDKN1A	Cyclin-dependent kinase inhibitor 1A (p21, Cip1)	−1.92	−1.05	−1.21	1.04
Hs.326035	NM_001964	EGR1	Early growth response 1	−2.88	−2.14	−2.79	−1.69
Hs.93177	NM_002176	IFNB1	Interferon, beta 1, fibroblast	−2.34	−1.46	1.14	−2.27
Hs.406266	NM_000189	HK2	Hexokinase 2	−2.5	−1.21	−1.43	−3.06

1 The complete list of genes analyzed using the Human p53 Signaling Pathway RT2 Profiler PCR Array (SABiosciences, Valencia, CA) includes: APAF1, ATM, ATR BAI1, BAX, BCL2, BCL2A1, BID, BIRC5, BRCA1, BRCA2, BTG2, CASP2, CASP9, CCNB2, CCNE2, CCNG2, CCNH, CDK1, CDC25A, CDC25C, CDK4, CDKN1A, CDKN2A, CHEK1, CHEK2, CRADD, DNMT1, E2F1, E2F3, EGR1, EI24, ESR1, FADD, FASLG, FOXO3, GADD45A, GML, HDAC1, HK2, IFNB1, IGF1R, IL6, JUN, KRAS, PIDD, MCL1, MDM2, MDM4, MLH1

MSH2, MYC, MYOD1, NF1, NFKB1, TP53AIP1, KAT2B, PCBP4, PCNA, PPM1D, PRC1, PRKCA, PTEN, PTTG1, RB1, RELA, RPRM, SESN1, SESN2, SIAH1, SIRT1, STAT1, TADA3, TNF, TNFRSF10B, TNFRSF10, TP53, TP53BP2, TP73, TP63, TRAF2, TSC1, WT1, XRCC5.

For CDKN1A, a slightly different picture emerged when using CXCR3 expressing cells as a baseline, compared to the parent HEK293 cells. While all cell lines showed some level of down-regulation compared to HEK293 cells, US28 was virtually identical to CXCR3 (1.04-fold change). In contrast, US27 cells exhibited a nearly 2-fold down-regulation of CDKN1A, which was nearly ablated in cells expressing either US27-DAY or US27/XR3CT. This decrease in gene expression correlated to a modest reduction in p21 protein levels compared to HEK293, US28, or CXCR3 cells (Fig. 4C). Since p21 is a negative regulator of cell cycle progression [21], [22], down-regulation of this protein by US27 supports our observations that the US27 protein conveys a proliferative and survival advantage. Further analysis of the possible interaction between US27 and the p21 signaling pathway may provide valuable insights into the mechanistic basis for this proliferative effect.

Some changes were notable because of a significant impact by only one of the GPCR examined. Cells expressing US28 had a dramatic increase in TNF (tumor necrosis factor) expression (4.41 fold) compared to the other cells lines. IFNB1, (interferon-β) was found to be up-regulated by 2-fold in cells expressing the US27/XR3CT mutant, but not by US27 or US27-DAY. However, these changes could not be confirmed at the protein level, and no TNF or IFNB1 was detected in the supernatants of any of the cell lines by ELISA (data not shown).

Another interesting change was the significant increase in expression of ESR1, the estrogen receptor, in cells expressing either the US27-DAY (3.02-fold change) or US27/XR3CT mutant (5.18 fold change), whereas expression was down-regulated in cells expressing either US27, US28, or CXCR3. In contrast, the WT1 (Wilms tumor) gene, a tumor suppressor implicated in childhood nephroblastoma [23], [24], was significantly down-regulated in cells with US27-DAY (−4.49-fold) with little impact from wild type US27 or US27/XR3CT, suggesting that there may be negative regulation of WT1 that is blocked by US27 and mediated through signaling involving the DRY box. Although US28 has previously been implicated in tumorigenesis [25], [26], future studies will be necessary to determine whether the US27 gene might play a role in tumor formation in either breast, kidney, or other forms of cancer.

In summary, we have shown that expression of HCMV US27 can promote cell proliferation, and that this effect requires both the DRY box motif and the carboxy terminal intracellular domain (CTD). While US27 has not yet been associated with any constitutive signaling pathway [27], our findings suggest that the viral receptor does likely associate with intracellular signaling proteins, such as p21 or Sestrin-2, and that the DRY box and CTD are important for these interactions. The requirement for the DRY box and CTD was less clear in protection from apoptotic stimuli, where the protective effect of US27 was diminished, but not completely abrogated, by the loss of either domain. Additional work is needed to fully understand the functions and signaling capabilities of this viral receptor, including whether these proliferative and pro-survival effects might play a possible role in tumorigenesis.

## Supporting Information

Table S1 **Gene expression analysis of US27 compared to HEK293.** RNA was extracted and expression levels of 84 genes involved in the p53 signaling pathway were analyzed using the p53 RT^2^ Profiler PCR Array. Raw data and analysis for three biological replicates of 293-US27 cells compared to values for the parent HEK293 cells is shown in this supplementary table.
(XLS)

Table S2 **Gene expression analysis of US27-DAY compared to HEK293.** RNA was extracted and expression levels of 84 genes involved in the p53 signaling pathway were analyzed using the p53 RT^2^ Profiler PCR Array. Raw data and analysis for three biological replicates of 293-US27-DAY cells compared to values for the parent HEK293 cells is shown in this supplementary table.
(XLS)

Table S3 **Gene expression analysis of US27/XR3CT compared to HEK293.** RNA was extracted and expression levels of 84 genes involved in the p53 signaling pathway were analyzed using the p53 RT^2^ Profiler PCR Array. Raw data and analysis for three biological replicates of 293-US27/XR3CT cells compared to values for the parent HEK293 cells is shown in this supplementary table.
(XLS)

Table S4 **Gene expression analysis of US28 compared to HEK293.** RNA was extracted and expression levels of 84 genes involved in the p53 signaling pathway were analyzed using the p53 RT^2^ Profiler PCR Array. Raw data and analysis for three biological replicates of 293-US28 cells compared to values for the parent HEK293 cells is shown in this supplementary table.
(XLS)

Table S5 **Gene expression analysis of CXCR3 compared to HEK293.** RNA was extracted and expression levels of 84 genes involved in the p53 signaling pathway were analyzed using the p53 RT^2^ Profiler PCR Array. Raw data and analysis for three biological replicates of 293-CXCR3 cells compared to values for the parent HEK293 cells is shown in this supplementary table.
(XLS)

Table S6 **Gene expression analysis of US27 compared to CXCR3.** RNA was extracted and expression levels of 84 genes involved in the p53 signaling pathway were analyzed using the p53 RT^2^ Profiler PCR Array. Raw data and analysis for three biological replicates of 293-US27 cells compared to values for the 293-CXCR3 cells is shown in this supplementary table.
(XLS)

Table S7 **Gene expression analysis of US27-DAY compared to CXCR3.** RNA was extracted and expression levels of 84 genes involved in the p53 signaling pathway were analyzed using the p53 RT^2^ Profiler PCR Array. Raw data and analysis for three biological replicates of 293-US27-DAY cells compared to values for the 293-CXCR3 cells is shown in this supplementary table.
(XLS)

Table S8 **Gene expression analysis of US27/XR3CT compared to CXCR3.** RNA was extracted and expression levels of 84 genes involved in the p53 signaling pathway were analyzed using the p53 RT^2^ Profiler PCR Array. Raw data and analysis for three biological replicates of 293-US27/XR3CT cells compared to values for the 293-CXCR3 cells is shown in this supplementary table.
(XLS)

Table S9 **Gene expression analysis of US28 compared to CXCR3.** RNA was extracted and expression levels of 84 genes involved in the p53 signaling pathway were analyzed using the p53 RT^2^ Profiler PCR Array. Raw data and analysis for three biological replicates of 293-US28 cells compared to values for the 293-CXCR3 cells is shown in this supplementary table.
(XLS)
